# Rapidly progressive gas-forming infection involving the spine as a life-threatening fatal condition : a case report

**DOI:** 10.1186/s12891-021-04589-8

**Published:** 2021-08-16

**Authors:** Hong Jin Kim, Ji-Hyun Ryu, Hyung-Youl Park, Sang-Il Kim, Dong-Gune Chang

**Affiliations:** 1 Spine Center and Department of Orthopedic Surgery, College of Medicine, Inje University Sanggye Paik Hospital, Inje University, 1342, Dongil-Ro, Nowon-Gu, 01757 Seoul, Republic of Korea; 2 Department of Orthopaedic Surgery, College of Medicine, The Catholic University of Korea, Seoul, Korea

**Keywords:** Gas-forming infection, Necrotizing fasciitis, Rapid progression, Spine, Epidural space, *K. pneumoniae*

## Abstract

**Background:**

Gas forming infection of the spine is a consequence of vertebral osteomyelitis, necrotizing fasciitis, or a gas-forming epidural abscess, which is very rare and fatal conditions. This is the rare case of necrotizing fasciitis that rapidly progressed from the lumbar area to upper thoracic area.

**Case presentation:**

A 58-year-old male complained of lower back pain with fever and chills. The patient had a history of uncontrolled diabetes mellitus without diabetic medication over the previous 3 months, and he had received several local injections around the lumbar area. Laboratory data revealed white blood cell count of 19,710 /mm^3^, erythrocyte sedimentation of 40 mm/h, and C-reactive protein of 30.7 mg/L. Radiological findings revealed a small amount of air bubbles in the paraspinal area and lumbar epidural spaces. The patient refused emergency surgery and was discharged from the hospital. The patient re-visited the emergency department two days after discharge complaining of more severe back pain with persistent fever, and his vital signs had deteriorated, with low blood pressure and tachycardia. *K. pneumoniae* was isolated in cultures from ultrasound-guided aspirates and peripheral blood. The follow-up radiographs revealed aggressive dissemination of innumerable air bubbles from the lumbar area to the T5 level. The patient underwent emergent decompressive laminectomy and debridement of infected paravertebral fascia and musculature. Despite intensive care for deteriorated vital signs and his back wound, the patient died on postoperative day 3 due to multi-organ failure.

**Conclusions:**

Necrotizing fasciitis involving the spine is a very rare disease with life-threatening conditions, rapid progression, and a high mortality rate. Therefore, prompt surgical treatment with a high index of suspicion is imperative to prevent potentially fatal conditions in similar extremely rare cases.

## Background

Gas forming infection (GFI) is a rare but rapidly progressive life-threatening infectious disease with radiological evidence of intra-spinal gas presented within the osseous, soft tissue, and/or neurological structures of the spine [1]. GFI involving the spine is especially rare, but it can be fatal due to conditions such as multi-organ failure [2]. Generally, GFI of the spine is a consequence of vertebral osteomyelitis, necrotizing fasciitis, or a gas-forming epidural abscess [3]. Since Bielecki et al. first described GFI in 1986, its distinct clinical features and significance have been recognized [4]. Although more commonly seen in the extremities, necrotizing fasciitis as a subgroup of GFI is very rare in the axial skeleton and has been mainly described in the form of case reports or case series, which are only reported 4 cases [1–5].

After 34 years from the initial description of GFI from Bielecki et al., the case of necrotizing fasciitis with gas-containing spinal epidural abscess across lumbar area to upper thoracic area has not been reported yet [3]. To the best our knowledge, this is the first case of fatal necrotizing fasciitis with rapid progression from the lumbar area to the upper thoracic area.

## Case report

**Fig. 1:**
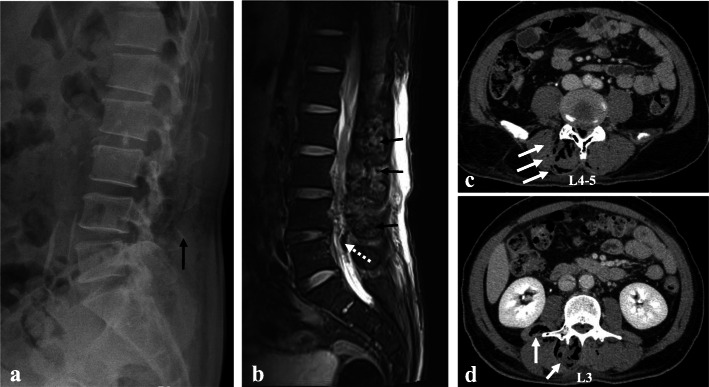
Lateral radiographs (**a**) showing a small gas-forming lesion around the lumbar paravertebral area (black arrow). T2-weighted sagittal magnetic resonance imaging (**b**) showing multifocal fluid pockets containing air bubbles in the paraspinal area (black arrows), whole lumbar area, and epidural space (white dotted arrow). Computed tomography (**c** and **d**) scan revealing subcutaneous emphysema in the right paravertebral muscles including the multifidus and quadratus lumborum, mainly in the right side (white arrows), as well as the lumbar spinal canal at L2-S1

A 58-year-old male visited the emergency department complaining of lower back pain, fever, and chills. The patient had a history of uncontrolled diabetes mellitus without diabetic medication during the previous 3 months, and he had received injection around the lumbar area in private clinics once a month for 3 months. An initial evaluation revealed a temperature of 38.1 °C, relatively stable hemodynamics (blood pressure 126/80mmHg, heart rate 109/min, and SatO2 96 %) and alert mentality. Physical examination demonstrated no infectious signs on the patient’s back, including fluctuation, erythema, or local heat, although local tenderness was noted at the right dominant lumbar paravertebral area. The neurological examination was within the normal limit without pathologic reflexes. Laboratory data revealed a white blood cell count of 19,710 /mm^3^ (92.7 % segmented neutrophils), erythrocyte sedimentation (ESR) of 40 mm/h, and C-reactive protein (CRP) of 30.7 mg/L. Initial assessment of diabetes mellitus showed Hb A1c of 13.5 %, glycated albumin of 53.4 %, and glucose 329 mg/dL. The other laboratory data are represented in Table 1. Laboratory risk indicator for necrotizing fasciitis (LRINEC) score was 9, which implied high suspicion for necrotizing fasciitis. The plain radiographs showed a small gas-forming lesion around the lumbar paravertebral area (Fig. 1a). Magnetic resonance imaging (MRI) showed multifocal fluid pockets containing innumerable air bubbles in the paraspinal area, whole lumbar area, and epidural space in T2-weighted sagittal view (Fig. 1b). Computed tomography (CT) scan revealed subcutaneous emphysema in the right paravertebral muscles including the multifidus and quadratus lumborum, mainly in the right side, as well as in the lumbar epidural space at L2-S1 (Fig. 1c and d). Ultrasound-guided aspiration was performed in the back area for evaluation of the infectious focus, and empirical antibiotics (piperacillin 4 g/tazobactam 0.5 g every 6 h) were immediately started to cover gram-positive and gram-negative bacteria. An emergent operation for abscess drainage and debridement of the infected paravertebral fascia and musculature was recommended, but the patient refused the surgical intervention. Unfortunately, the patient was discharged against medical advice and prescribed with only antibiotic drug (cefadroxil 500 mg BID [bis in die] for 5 days).

**Table 1:** Clinical laboratory data on the patient with necrotizing fasciitis involving the spine

Variables	Initial evaluation	Re-visit evaluation	Reference range
Hemoglobin (g/dL)	12.3	12.8	13–17
White-cell count (/µL)	19,710	5,450	4000–10,000
Platelet (/µL)	213,000	46,000	150,000–400,000
PT INR	1.19	1.37	0.9–1.2
aPTT (sec)	41.0	35.5	28.0–45.0
BUN (mg/dL)	16.1	38.5	7–25
Creatinine (mg/dL)	0.77	0.91	0.5–1.2
Albumin (g/dL)	2.9	2.1	3.5–5.3
AST/ALT (IU)	54/86	47/49	0 ~ 40
Sodium (mEq/L)	131	130	135–145
Potassium (mEq/L)	4.3	4.2	3.5–5.5
Chloride (mEq/L)	99	100	98–110
CRP (mg/dL)	30.7	25.0	< 0.3
Glucose (mg/dL)	329	269	70–110
HbA1c (%)	13.5	-	4.3–6.3
Glycated albumin (%)	53.4	-	11–16
Lactic acid in ABGA (mmol/L)	-	6.9	0.7–2.1

Laboratory risk indicator for necrotizing fasciitis (LRINEC) score was 9 in initial evaluation, which implies high suspicion for necrotizing fasciitis

*BUN* Blood urea nitrogen; *AST* Aspartate aminotransferase; *ALT* Alanine aminotransferase, *ABGA* Arterial blood gas analysis

**Fig. 2:**
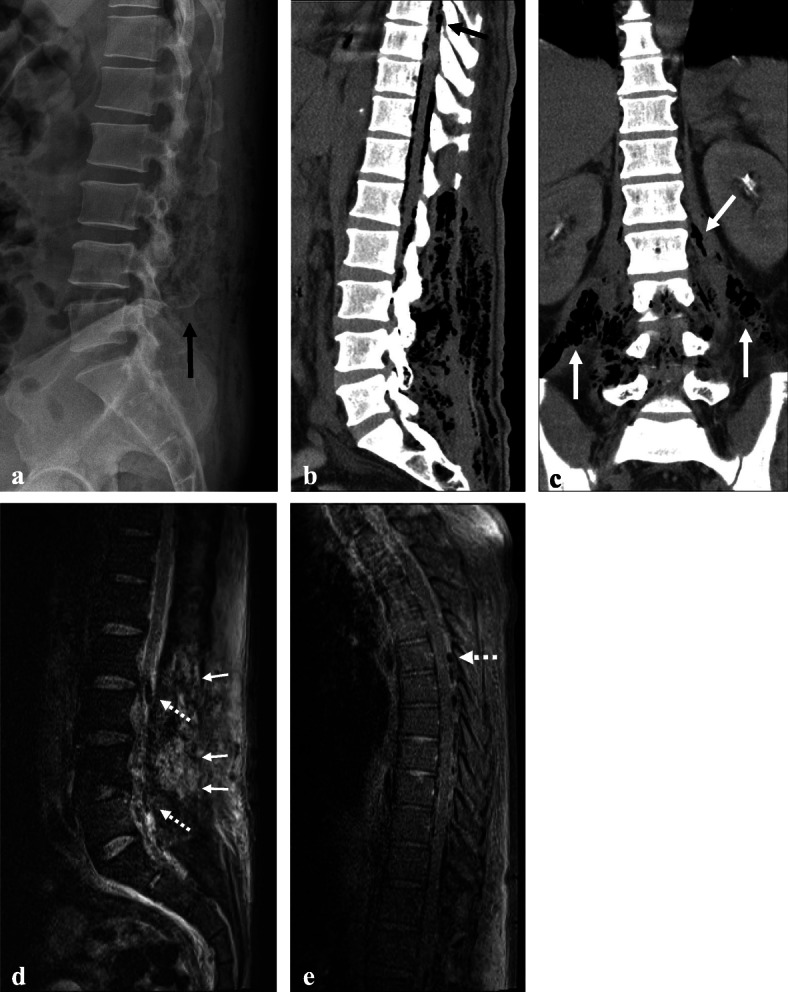
Lateral radiographs (**a**) showing an aggressive gas-forming lesion around the lumbar paravertebral area (black arrow). Sagittal and coronal computed tomography (**b** and **c**) scans showing aggravating subcutaneous emphysema in the psoas muscle (white arrows) and paravertebral muscle as well as in the lumbar epidural space. Sagittal magnetic resonance imaging (**d**) showing aggressive dissemination of multifocal fluid pockets containing innumerable air bubbles in the paraspinal area, whole lumbar area (white arrows), and epidural space (white dotted arrows), which disseminated into the T5 level (white arrow) (**e**)

**Fig. 3:**
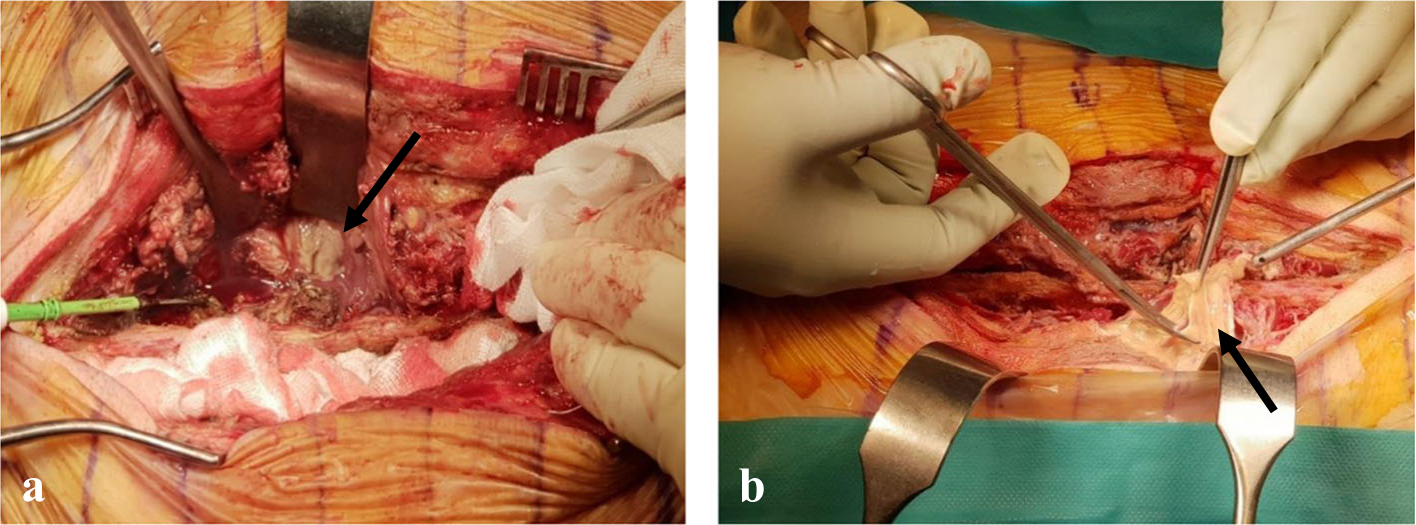
Intra-operative findings (**a** and **b**) showing a large amount of pus-like exudate along the superficial and deep fascia as well as devitalized color change along the membrane of fascia (black arrows)

Two days later, the patient returned to the emergency department complaining of more severe back pain and persistent fever (38.4 °C). His vital signs and laboratory data had deteriorated (Table 1). He was hypotensive (81/49mmHg), tachycardic (heart rate 141/min) and high level of serum lactic acid (6.9 mmol/L) in arterial blood gas analysis, implying a septic condition. Cultures from the previous aspirates and peripheral blood were isolated *Klebsiella pneumoniae (K. pneumoniae)*. Antimicrobial susceptibility test of *K. Pneumoniae* showed all sensitive except for ampicillin and targeted antibiotics (ceftriaxone 2 g) were administered every 24 h in consultation with Department of Infectious Disease. The follow-up radiological examinations (plain radiograph, CT, and MRI) showed aggressive dissemination of innumerable air bubbles from the whole lumbar area to the T5 level (Fig. 2). Especially, innumerable air bubble was observed throughout bilateral psoas muscles in the coronal view of CT, which showed bilateral necrotizing fasciitis of the psoas muscles (Fig. 2c). The patient underwent emergent decompressive laminectomy for abscess drainage from L2 to S1 and debridement of the infected paravertebral fascia and musculature using metzenbaum scissor and rongeur. The intra-operative findings showed more severe and extensive than radiological findings, which disseminated to epidural region, ligament flavum, all muscle layer, and just beneath skin region. A large amount of dishwater-like exudate along the superficial fascia, devitalized color change along the membrane of fascia, and purulence was also observed in the epidural spaces from L2 to S1 (Fig. 3). We would have planned second-stage operation for psoas muscle fasciitis after patient’s condition improved with use of antibiotics in Intensive Care Unit (ICU). Therefore, we applied vacuum-assisted closure (VAC) for potential residual infection and wound care after massive irrigation (dilute iodine solution, normal saline, and saline with added ceftriaxone by simple syringe irrigation) and debridement of the devitalized tissue. *K. pneumoniae* was detected in cultures obtained from intra-operative samples. Despite intensive care for his deteriorated vital signs and back wound, the patient did not recover and died on postoperative day 3 due to multi-organ failure.

## Discussion and conclusions

Necrotizing fasciitis is a surgical diagnosis characterized as a rapidly progressive deep soft tissue infection associated with the signs of sepsis and multi-organ failures [6]. Its pathogenesis involves rapid inflow of infected cells to the fascial plane, muscle cell death, and ischemia from thrombus formation in the microcirculation [6–8]. Poor blood supply in infected muscle together with lower number of immune cells in the layer allows for progressive spread of infection, which lead to devastating consequences in the surrounding muscles [9, 10]. The surgical features of necrotizing fasciitis are dishwater-like exudate, fluid in the wound, lack of fresh bleeding, and color change of the fascia. The term necrotizing fasciitis was first described by Wilson in 1952, with these surgical characteristics, and approximately 500 to 1,500 cases involving the lower extremities are reported per year, with high mortality rates. Necrotizing fasciitis involving the spine, however, was reported in only 4 cases in the literature, and all four patients had uncontrolled diabetes mellitus, as in our case [2, 7–9].

Giuliano et al. clinically classified necrotizing fasciitis into two types: type I (mixed infection caused by aerobic and anaerobic bacteria) and type II (mono-microbial infection caused by group A streptococcus) [10]. Type I necrotizing fasciitis predominantly involves microbes such as *Staphylococcus aureus (S. aureus)*, streptococci, enterococci, and *Escherichia coli (E. coli)*. Moreover, this form of necrotizing fasciitis most commonly occurs after surgical procedures and in patients with diabetes and/or peripheral vascular diseases [7]. In our case, patient had uncontrolled diabetic mellitus and several injections history at private clinics. Injection history could also provide better understanding of necrotizing fasciitis as one of infective risk factors. However, we have limited information except for injection around lumbar area by pain medicine specialist at private clinics once a month for 3 months. Although minimal invasive spinal injection is one of the common treatments for back pain in private clinic settings, it would be developed fatal conditions in patients with diabetes and/or immunosuppression. Therefore, the patient’s conditions such as diabetes mellitus, immuno-competent status, neurological disorders, and severe cardiovascular disorders should be thoroughly assessed prior to the planned injection therapy. Type II necrotizing fasciitis can occur in any patient group, and streptococcus is typically detected [11]. Regarding the 4 previously reported cases of necrotizing fasciitis involving the spine, one was type I necrotizing fasciitis in which a combination of *E. coli* and *S. aureus* was isolated, and three cases were type II necrotizing fasciitis in which *K. pneumoniae* and *S. aureus* were isolated. In agreement with the presented case, *K. pneumoniae* and *S. aureus* are common isolated microbes of necrotizing fasciitis involving the spine, in four cases. Furthermore, all previously reported cases of necrotizing fasciitis involving spine have shown a 100 % mortality rate. Therefore, *K. pneumoniae* and *S. aureus* could be considered as the important microbes in necrotizing fasciitis involving the spine, which showed poor prognosis.

Clinical presentations for necrotizing fasciitis are non-specific and difficult to differentiate from other infectious diseases [1–3]. The presence of crepitation, swelling, or erythema is a clinical sign of necrotizing fasciitis [7, 8]. Moreover, clinical manifestations like bullae formation, skin necrosis, and edema are important to the sign of rapid worsening condition [9, 10]. From the radiological evaluation, gas formation along the paraspinal muscle is a common characteristic of necrotizing fasciitis involving the spine, which is specifically measured by CT [8, 12–15]. The aggressive nature of necrotizing fasciitis involving the spine was indicated by gas-forming lesion from our case, which showed rapid progression from lumbar area to the upper thoracic area within 1 week. Therefore, surgeons should recognize that gas-forming lesions have the characteristic of rapid progression and can result in fatality if untreated.

The surgical treatment for necrotizing fasciitis must include removal of all devitalized and infected tissue. Autopsy was considered as confirmation test by intraoperatively taken tissue sample, which provided better illustration of necrotizing fasciitis. However, in our case, biopsy was not performed because 9 of LRINEC score and radiological findings were consistent with necrotizing fasciitis. Wound closure is not recommended because of poor wound healing and devitalized fascia plane [16]. Wound VAC with thorough debridement is one of the important treatments of residual invisible infected tissue. Serial dressing after surgical debridement is the only surgical option before wound VAC care, but recent advancements in VAC overcome the limitations of open wound care. The mortality rate, however, did not decrease over time with recent advances in antibiotics and methods of wound care, including VAC [8]. From our experience with necrotizing fasciitis, prompt surgical treatment upon diagnosis based on gas-forming radiological findings is the most important factor to reduce the mortality rate.

The prognosis of necrotizing fasciitis is extremely poor because of its rapidly destructive progression from local necrosis to systemic dissemination to sepsis [12]. Despite emergent surgical intervention, necrotizing fasciitis including from extra-axial to axial skeleton showed 34 % of mortality rate and associated with septic conditions [3, 5]. For the cases of necrotizing fasciitis in axial skeleton, Beit Ner et al. reviewed gas-forming infections of the spine and reported a 100 % mortality rate for necrotizing fasciitis [3]. Similar to our case, the patient with necrotizing fasciitis suffered from the aggressive and rapidly progressing nature and died. Based on the outcomes from these previous cases of necrotizing fasciitis, surgical intervention might be considered a meaningless choice for treatment. However, based on the outcomes of necrotizing fasciitis outside the axial skeleton, early diagnosis, emergency surgical treatment, and broad-spectrum antibiotics offer a chance for increased survival rates [1, 13–16]. Although diagnosis is challenging because of difficulty differentiating necrotizing fasciitis from other infections like cellulitis, a gas-forming lesion involving the epidural space should produce a high index of suspicion, as demonstrated in our case [13]. Surgical intervention is the cornerstone in the management of necrotizing fasciitis because early debridement is the only factor that can lower the mortality rate [17]. Therefore, prompt surgical treatment before dissemination to other organs such as psoas muscle in retroperitoneal space is critical for decreasing the mortality rate of necrotizing fasciitis involving the spine.

In conclusion, necrotizing fasciitis involving the spine is a very rare disease with life-threatening conditions, rapid progression, and a high mortality rate. Therefore, Prompt surgical treatment with a high index of suspicion is imperative to prevent potentially fatal conditions in similar extremely rare cases.

## Data Availability

Data sharing is not applicable to this article as no datasets were generated or analyzed during the current study.

## Abbreviations

GFI: Gas forming infection
ESR: Erythrocyte sedimentation
CRP: C-reactive protein
LRINEC: Laboratory risk indicator for necrotizing fasciitis
MRI: Magnetic resonance imaging
CT: Computed tomography
BID: bis in die
*K. pneumoniae*: *Klebsiella pneumoniae*
S. aureus: *Staphylococcus aureus*
*E. coli*: *Escherichia coli*
ICU: Intensive Care Unit
VAC: Vacuum-assisted closure
